# Perceptions of Cross-Cultural Challenges and Successful Approaches in Facilitating the Improvement of Equine Welfare

**DOI:** 10.3390/ani13111724

**Published:** 2023-05-23

**Authors:** Suzanne Rogers, Natasha Y. P. Lee, Jo White, Catherine Bell

**Affiliations:** 1 Equine Behaviour and Training Association, Surrey GU8 6AX, UK; 2 Human Behaviour Change for Life CIC, Norfolk NR9 4DE, UK; 3 Asia Animal Happiness Consultancy Puchong, Selangor 447180, Malaysia

**Keywords:** equine welfare, working equines, cross-culture, perceptions, animal welfare

## Abstract

**Simple Summary:**

Many projects that aim to improve the welfare of equids worldwide involve people from different countries and cultures working together. Given that professionals involved with multi-stakeholder projects often work cross-culturally, this study examined their experiences regarding the challenges involved and their reflections on how to work in a culturally sensitive way. Semi-structured interviews were conducted with 14 participants working in a total of 29 countries and analysed using thematic analysis. Key themes emerged from the responses to questions covering the areas of perceptions of animal welfare, challenges working cross-culturally and embracing cultural sensitivity. Previous works have highlighted the importance of shared linguistic knowledge, interpersonal skills and cultural knowledge, and these elements also emerged in this research. As well as providing insights into the challenges of working cross-culturally, the findings of this study have enabled the development of suggestions for how this work could be taken forward in a practical way to be of use to professionals in this sector.

**Abstract:**

Projects that aim to improve the welfare of equids worldwide usually involve people from different countries and cultures working together. Given that professionals involved with multi-stakeholder projects often work cross-culturally, this study examined their experiences regarding the challenges involved in, and their reflections on, how to work in a culturally sensitive way. Semi-structured interviews were conducted with 14 participants working in a total of 29 countries and analysed using thematic analysis. Key response themes emerged from the responses to questions covering the areas of perceptions of animal welfare, challenges working cross-culturally and embracing cultural sensitivity. The overriding theme regarding perceptions of animal welfare was that of barriers to animal welfare, under which emerged the subthemes of limited financial and material resources, limited understanding of the tenets of animal welfare, and attachment to traditional medicines and practices. Exploring the key challenges resulted in two themes: challenges regarding the local context and etiquette, and those regarding working with different stakeholders. Considering cultural sensitivity, again, two themes emerged: the importance of trust and respect, and of working with local partners. Previous works have highlighted the importance of shared linguistic knowledge, interpersonal skills and cultural knowledge, and these elements also emerged in this research. As well as providing insights into the challenges of working cross-culturally, the findings of this study have enabled the development of suggestions for how this work could be taken forward in a practical way to be of use to professionals in this sector.

## 1. Introduction

There are four major organisations based in the UK [1], and similar organisations in other countries, that are working internationally with the aim of improving the welfare of working equines. Many of the most significant funding organisations regarding this work are acting cross-culturally, as they manage and fund projects in different countries to those in which the key staff are based. 

There are an estimated 100 million equids in low- to middle-income countries, most of which are working animals providing income and support for their owners and communities [2]. Working equines are often owned by people with low socioeconomic status and, as such, are particularly important to marginalised and vulnerable groups (reviewed in [3]). Owners and caregivers are often unable to afford to manage their animals in a way that safeguards their welfare (reviewed in [3]). A study from nine low- to middle-income countries found that 90% of equids were suffering from hoof and limb issues, and 85% were underweight, with lameness, poor body condition, wounds, disease, parasites and dental problems also commonly reported ([4,5,6] and reviewed in [3]). 

Projects aiming to improve the welfare of working equines often involve many stakeholders such as local veterinarians, local project staff, project managers (often not local), the authorities, veterinary authorities, tourists, equine owners and people who rent equines. Such projects, therefore, often involve people managing or working with others who belong to a different culture, i.e., working and communicating cross-culturally. Such cross-cultural working can pose challenges regarding clarity of communication and effectiveness. This paper aims to explore the experiences and recommendations of several people who work in this way. 

The term “culture” is used to describe the collective social behaviour, norms and institutions found in human societies and includes collective knowledge, beliefs, arts, laws, capabilities and habits of individuals [7]. The Cambridge English Dictionary states that culture is *“the way of life, especially the general customs and beliefs, of a particular group of people at a particular time.”* [8]. Therefore, although culture is often thought of as being shared between people in the same geographic location, in practice, people might identify more strongly, in terms of culture, with people sharing their religion, socioeconomic status or scientific discipline [9] than with people from the same region or country. 

Saville-Troike’s work [10] on cross-cultural communication demonstrated that, for successful communication to take place between people of different cultures, a person must have the appropriate linguistic knowledge, interaction skills and cultural knowledge. Building on this work, specifically with regard to qualitative interview research, Stanton argued that, to avoid misunderstandings, the interviewer must try to walk in the other person’s shoes [11]. This is relevant to the present study not only in terms of communication between the professionals working cross-culturally with equid owners but also regarding the cross-cultural communication between the researcher and interviewees.

Perspective taking is the process by which we can understand the viewpoint of another person. Learning to do so has been considered, to some extent, to be a normal part of child development [12]. However, the degree to which we deliberately implement perspective taking when working cross-culturally can impact the success of those interactions. For example, Todd et al. [13] found that perspective taking was successful in reducing racial bias and improved the interpersonal experience of the interaction. Similarly, perspective taking during mediation was able to reduce conflict and improve relations between groups [14]. In a study of intergroup threat, opposition to policies that were perceived as threatening and overly beneficial to others was reduced in the experimental group who experienced perspective taking [15]. However, there are also cultural differences in perspective taking and empathic concern, whereby countries measuring more highly on empathy (as distinct from, but related to, perspective taking) were also found to have higher levels of collectivism and prosocial behaviour [16]. 

Over the past 15 to 20 years, the approaches used by international non-governmental organisations (iNGOs) have changed from predominantly reactive approaches, focusing on providing access to veterinary resources, to a proactive approaches, focusing on community engagement and prevention [17]. This shift has perhaps driven a change in the way that iNGOs, local partners and working equid owners interact with each other and has arguably made the need for the understanding of cross-cultural working even more important. However, as also recognised by Sinclair and colleagues [18], apart from lessons learned by trial and error and personal experiences informally shared regarding working cross- culturally in animal welfare projects, there is a gap in the research to support the challenging task of running impactful projects internationally. Aside from the work of Sandøe and colleagues [19], there is a lack in the scientific literature exploring the ethical aspects of cultural differences regarding working equid welfare. In addition, much of the research published regarding the welfare challenges for working equids and the impact of projects to address those challenges (for example, [20]) has not covered the interpersonal aspects between the people working on those projects; this paper seeks to address this gap. 

This qualitative study consisted of a series of semi-structured interviews with experienced professionals working in several different roles with the common goal of improving the welfare of working equines. The interviews were used to provide insight into the challenges faced, and successful approaches used, by staff working cross-culturally in iNGOs. 

There were four key objectives of this study: first, to gain an understanding of the similarities and differences regarding how animal welfare is considered by people from different cultures; second, to gain an understanding of the challenges associated with working with stakeholders across different cultures; third, to gain insight into some ways that professionals aim to work in a culturally sensitive way; and fourth, to synthesise information in a way that will be useful for people when planning and implementing programmes to improve equine welfare across different cultures. 

Despite the variations of the contexts and locations of the interviewees included in this study, there were key similarities in their perceptions of the crucial points to consider when working cross-culturally. Participants’ responses were considered by taking into account Saville-Troike’s elements [9] for effective cross-cultural communication. Although there are academic networks focusing on equine welfare internationally, few studies have synthesised the experiences of project managers in the field. To the authors’ knowledge, this is the first study in the sector to examine the cross-cultural interpersonal aspects of equine welfare professionals. It is hoped that the collective experiences documented can aid everyone working in the field when planning, implementing, assessing or even just visiting projects that aim to improve equine welfare. 

## 2. Materials and Methods

Interviews were conducted throughout January and February 2023 with a total of 14 people involved with projects concerning working equines in 29 countries (Sri Lanka, Indonesia, Thailand, Cambodia, Nepal, Costa Rica, Nicaragua, Honduras, Panama, Guatemala, Colombia, Argentina, Chile, Mexico, Haiti, Cuba, Hawaii, Senegal, the Gambia, Ghana, Kenya, Burkina Faso, Ethiopia, South Africa, Lesotho, Zimbabwe, Jordan, Pakistan and India; note that some interviewees worked in several countries). Interviewees were selected from the authors’ network and resulting recommendations. All interviewees are currently heavily involved with equine welfare projects and work across cultures in different ways; some are native to one region and work with equid owners in another region, some share nationality with the equid owners but are employed by funding organisations from a different region, some manage projects in several countries and so on. 

The criteria for inclusion were that the interviewee had to be professionally involved with working equines and that there had to be a cross-cultural nature (where culture was initially considered to be a shared native country) of that work. Through this form of sampling, a volunteer sample of participants was recruited. Note that communications were conducted in line with the processes outlined during the ethical review approval procedure (see the Institutional Review section at the end of this paper). After 14 interviews, data saturation was reached, with no new themes emerging using thematic analysis. 

Interviews were arranged using email and took place online using Zoom 5.11.1 (6602) video conferencing software. For the subsequent transcriptions, all interviews were recorded and ranged in length from 18 min 06 s (Participant 12) to 45 min 56 s (Participant 13). Participants received a briefing document detailing information about the project and regarding consent before the interviews were conducted. Participants gave their verbal consent to the process at the beginning of each interview. 

Participants were asked five questions on the theme of their experience working cross-culturally in their professional roles. The interviews were semi-structured, so the question script was flexible, and clarification was sometimes requested. Semi-structured interviews were chosen as the qualitative research tool because they provide a flexible technique for small-scale research [21], provide useful data from small sample sizes and allow thematic analysis of the qualitative data [22].

The interviews were transcribed using an automated online service and edited to remove duplicate words, the phrases “I know”, “You know”, “So”, “Uh” and “Um” and to change the spelling from American English to British English. The names of any organisations were also edited out to remove any conflicts of interest and to maintain some anonymity. The grammar was edited throughout; for example, adding punctuation to help the readability, especially ‘full stops’, because the automatic transcription programme often created very long sentences. These changes were made whilst listening to the audio recording to ensure that the meaning was not changed by the editing. Participants were assigned codes P1 to P14 according to the order in which they were invited for interview. Thematic analysis was conducted following Braun and Clarke’s six-stage process [23]. As also described by Rogers and Bell [24], Stage 1 (familiarisation) involved reading the transcripts and listening to the recordings to remind the researcher of the interview contents. Stage 2 (coding) was performed on the transcripts from the 14 interviews to facilitate the generation of themes. This stage was performed manually using the highlighter function in MS Word (Microsoft^®^ Word for Microsoft 365 MSO (Version 2209 Build 16.0.15629.20200) to colour similar concepts and phrases that were repeated throughout the interviews [25]. The documents were then clustered, putting similar contents together, to enable stage 3 (generation of themes). In stage 4, these draft themes were reviewed to enable themes to be combined or separated. In stage 5, the themes were then defined and named before stage 6 in which they were written up into a narrative as the research results. The process was conducted by the lead researcher with support from a second person. The transcripts were all read again to extract quotes that were especially relevant to the emerging narrative. Finally, the extracted quotes were arranged (in a Word document) so that the most representative quotes could be used in the write-up of the research paper.

## 3. Results

The interviews explored four key areas: the context of the participant’s work, perceptions of animal welfare across cultures, the challenges of working cross-culturally and embracing cross-cultural sensitivity. This section outlines and discusses the results of the thematic analysis that was used to examine the responses to questions in each of these four areas.

### 3.1. Exploring the Context of Participants’ Work

It was found that some interviewees work in the country that they are native to, some work in many countries in a region that they are native to, and some work in a country that they had no connection with before their current role. The nature of cross-cultural working was described, for example, some interviewees work in their native country for an iNGO based in another region of the world. Participants described projects that involve stakeholders with multiple nationalities, for example, regional or national project staff, people from the donor organisations, international staff and tourists. 

The participants described the nature of their role. The participants were all involved with projects aiming to improve the welfare of working equids: horses, ponies and mules used for activities such as the transport of people, goods and water; agricultural work such as ploughing; work in brick kilns and in the tourist industry (e.g., transport of tourists, tourist leisure riding and one participant was involved with a business providing rides for tourists rather than an equine welfare project). In addition, one interviewee was also working on a project regarding donkey slaughter and another on a project with equines used by the army. 

Using the term “culture” as an umbrella term to encompass nationality was found to be unsuitable, as it became clear that the term “culture” more accurately refers to people who share similar socioeconomic backgrounds, education, religion, language or areas of habitation. This meant that participants who work with equid owners with shared nationalities to themselves often found differences in their “cultures”, e.g., *“My case is quite particular because I’m a returnee from Cambodia. I grew up in France, so from my accent they consider me as a foreigner. When I say, “I’m Cambodian”, they say, “No, you’re not Cambodian, you’re Korean!” so I have already this barrier in the sense of language.”* (P6). Another participant outlined similar language challenges. Although also Sri Lankan, her primary language was English and not the predominant language, Singhalese. She had to translate all the veterinary terms from English into Singhalese in her head and reported that *“They think that I’m like struggling with myself, but it’s more that I’m struggling with the language.”* (P8). Finally, participants from the city described struggling to resonate with rural owners, even though they both came from the same country: *“We are all from the same country, so the culture is the same. But the lifestyle, the way of living, is very different—some areas are very rural, so the lifestyle, the way of living, is different from ours in the city*.” (P5).

### 3.2. Perceptions of Animal Welfare

All the participants used the terms “animal welfare” and “equine welfare” throughout the interviews. The interviewees seemed keen to share the contexts of the equine owners they work with, and P10’s quote was representative of the discussions regarding perceptions of animal welfare: *“I think the bigger problem isn’t that people can’t understand, it’s that if they don’t have money, they can’t do much better”.*

When exploring perceptions of animal welfare in the countries in which the participants work, the overarching theme that emerged was “barriers to welfare”. Within this overarching theme, three subthemes were nested that relate to the causes of these barriers: “limited financial and material resources”, “limited understanding of the tenets of animal welfare” and “attachment to traditional medicines and practices”. The first subtheme (limited financial and material resources) is best represented by the following quote: “*The carriage horse owners here are living in extreme poor economic conditions. They live next to the stable, not even a stable, an enclosure that is far from the hygiene and sanitations, where they also live, side to side with the cow, goat, the horses as well. In one tiny house, 10 or 15 people. They cannot fulfil their own welfare and that’s why it’s very difficult to get across the message about giving or providing welfare for their animals*.” (P9).

Regarding subtheme 2 (limited understanding of the tenets of animal welfare), it was found that there is a general shared understanding of equine health between project managers, equid owners and funders but that the welfare domains are not well understood. Examples of illustrative quotes for this theme include: *“You say welfare in Spanish, which is ‘bienstar’, and automatically people start thinking about health and they start talking about vaccines and dewormers. They don’t consider that horses are social creatures, have emotions, or can be in pain.”* (P11) and *“A lot of the owners certainly see animal welfare as a negative thing, thinking of it as an enforcement process or something that’s going to stop them using animals for their livelihoods.”* (P3). The interviewee involved with a riding tour company (P14) described a different context whereby the tour operator’s horses were well-looked after and resourced, but welfare was still not understood as a concept.

Regarding subtheme 3 (“attachment to traditional medicine and practices”), the participants mentioned challenges with traditional medicine and practices, that create a barrier to safeguarding welfare. For example, *“It was very difficult at the beginning for us to change their traditional practices to the new ones.”* (P7), and more specifically, *“Most have stopped using monkeys [monkey blood] to treat horses but they do use traditional medicine a lot—and also human medicines to treat ponies! They give 10 times human tablets, for example, which can cause huge problems.”* (P5) and *“A key issue is traditions passed down from generations; for example, urinating on the hoof to cure ‘spider bites’ or using beer to cure colic.”* (P11).

Under the main theme (“barriers to animal welfare”), some examples of controversial aspects of animal welfare and how they are interpreted between different cultures and countries were provided. P8 explained how people often think that, if they neuter their animal, it will affect the person responsible for the animal in the same way, and they will not be able to have children either. P4 explained that a key challenge for her organisation is that Thailand is predominantly Buddhist, posing some moral and ethical dilemmas regarding welfare. For example, Buddhists resist the concept of euthanasia, believing that animals should be allowed to die naturally: *“To me that’s a conflict because the animal was never allowed to live naturally, so why should it have to die naturally?”* She further explained that animals in the wild are different but, when in our care, we have the responsibility to prevent suffering. She went on to say *“It’s the whole idea of samsara—that all life forms are a circle—there’s rebirth, and reincarnation. I sometimes explain to owner that you could come back and get reborn as a pony so do you want to be treated like that?”.* P12 described challenges regarding the use of the word “welfare”: *“With some owners, we don’t use the word ‘welfare’ because they are afraid of activist groups that are trying to ban working animals […]—we talk about good management practices, behaviour, health, emotions of the horses.”* Some participants reflected on how the different ways horses are thought of from various ethical viewpoints affects the way they respond to the subject of animal welfare. For example, P3 explained that *“Some Ghanaians view animals very differently than we do—for example, they believe that animals have been given to us to be used and subsequently animal welfare is just not a widely discussed concept, animals are a means to an end. They don’t see animals as something to hurt deliberately or to treat with malicious intent, it’s more that there’s a lack of understanding about their sentience and their ability to feel emotions and pain.”*.

Despite the extensive barriers to animal welfare, all participants expressed throughout the interviews that the equine owners they work with, directly or through partners, are keen to learn and are open to discussions about equine welfare (whether or not explicitly termed “animal welfare”). P4’s quote illustrated this theme: *“When people actually know that something is affecting the animal badly, they are quick to ask how to do it differently? People do care”*.

Some participants reflected on the way different stakeholders navigate the different understandings of animal welfare. For example, P6 explained *“Our funding organisation is very respectful of the local communities and local NGO they’re working with. They give us the notion of what is welfare, and we cannot compare the welfare standard in Europe and the welfare standard in our community, in our country. Its too much to tell people to reach the standard in the sky when you are on the earth. [The organization] do not implement a ‘must do’ approach; they have their guidelines, and we can do as much as we can to reach that standard but they do not push us to reach a certain point. And I’m very appreciable for them because at a certain point, if you put too much, it will break and then everything will collapse. And this is very sensitive.”* P13 articulated that solutions require a systems-based approach to animal welfare, where the role of the working equine is recognised as providing an important service to the community and, therefore, that it is not only the owner’s responsibility but also that of local service providers, authorities, NGOs and other stakeholders.

### 3.3. Challenges Regarding Working Cross-Culturally

Perhaps the most representative reflections on this issue were articulated by P3: *“I think the biggest challenge for me in working cross-culturally is trying to separate out my own emotions from what I’m witnessing because it is so different to how we treat animals or how we see animals in our own culture. Trying to avoid judging because trying to put yourself in put in their shoes is incredibly difficult when I recognise that I have this massive white privilege, that I take for granted and I have so many things that they don’t have. […] Just trying to stay motivated and positive is really difficult when you’re faced with such an awful animal welfare but also human welfare that is restricting their access, their ability to be able to improve that animal welfare.”*.

Two key themes emerged regarding challenges in working cross-culturally: “challenges regarding understanding local context and etiquette” and “challenges regarding working with different stakeholders”. 

Theme 1, “challenges regarding understanding local context and etiquette”, was illustrated by P13, who described the importance of mapping out a project to ensure the local context is truly understood because the project will effectively be external support to an area that has its own social and economic structures. He also described how the way the project is framed is important: *“We explain that we are not here to tell you what you need to do but we are here to learn from you, to support what you are doing, to manage risks and potentially improve what is good”.* P13 also described the importance of understanding the cultural background regarding the history of working equines in the project area: *“In Latin America, the horse is seen, especially in the indigenous areas, as fairly new tool for the communities because before the introduction of motor vehicles equids were mainly used for the wealthy landowners. The indigenous communities usually had to transport things themselves. Since the beginning of the motor vehicles the use of horses for transport has been pushing into more into the indigenous rural communities more and more.”* P9 described how, in parts of Indonesia, the status of horse cart drivers has changed over the past 30 years. He said that 30 to 40 years ago, having a horse and cart was a source of pride and that they were relied on for transport. However, now, people in the community have motorbikes, cars and public transport, so things have changed, and being a cart driver has lost status. He described how the pandemic exacerbated this, as the tourist industry stopped, and now, the situation for horse cart drivers is one of abject poverty. 

P12 highlighted the need to identify the context regarding social cues even if working in your own country: *“The first visit is just to talk with them, not even to see the horses but to see what they expect for us from us and see how we can help. Sometimes we can’t help, but we can make a link with the municipality or with other people that maybe can help. The other thing, we have learned is not to impose our position. We try to facilitate the work and see if they can come up with solutions because otherwise it’s not sustainable.”*. 

The context regarding social structure is also relevant: *“Cultural sensitivity extends to being sensitive to […] things like their cultural structure—like the structure of families and communities and the roles within them. Some of the more rural places in Ghana have very strict roles and responsibilities and hierarchies, not just within the family but within the village. They’ll have village elders who they go to for advice. [..] Working those relationships out, being sensitive to them, and working with the structure, not against it, is really important.”* (P3).

P11 emphasised that the importance of not going into a community with a preconceived idea of what needs to be changed but rather working with the community to find out the context in terms of their issues, concerns, priorities and so on and how this approach can nurture trust, because you are helping them with an issue about which they are concerned. Regarding funders, one participant (P9) appreciated that a funder asked them to fill in an extensive questionnaire to understand the context of the working equine owners. 

The need to understand the local context was expressed by the majority of the interviewees and eloquently summarised by P13: *“It is important that equid welfare projects that are coming from donors in Europe or the USA have a good understanding of the social, cultural and economic context of the communities. […]. But we need to be very realistic about our expectations about what we can achieve—in a five- or ten-year project.”.* P13 also described how different stakeholders think along different timescales: *“Owners are thinking, day to day, about what they’re going to do tomorrow or what they’re going to do a couple of days later, local authorities are thinking in terms of two years or four years—the time that they are in office. […].”*.

Several interviewees expressed that it takes time to change behaviours and make an impact. For example, *“Sometimes it is not appreciated how slow it is to shift deeply held beliefs and attitudes. […] We are not talking a workshop or a, a pamphlet or an advert or a billboard, it takes long hours of discussions, contact, time, trust, and building a relationship.”* (P2). P7 described how sometimes the donors who live some distance from the project want to implement things that just are not practical within the project’s context, especially in the timeframe expected. He also described the challenges of working with funding organisations based in other countries: *“The organisations do understand when they have visited the communities. It is sometimes very difficult to convince them through a Zoom call but when you bring them into the field and provide the exposure with the communities it is very easy for them to understand.”*.

Other reflections on the theme of understanding the context included topics such as meeting etiquette and communication challenges. For example, P10 explained that she has experienced cultural challenges regarding behaviours associated with working in an effective team (e.g., being on time and prepared for meetings) and feels that a global professional culture is emerging as more people work cross-culturally. P10 also described how sometimes she must manage the expectations of the international project funding organisation. For example, in cases where the project had not progressed as hoped, she acted as an intermediary to ensure that the language used was clear to the staff on the ground. This had not always been the case due to cultural differences regarding the considered polite ways of framing things; sometimes, a more direct approach had been needed for the message to become clear. 

Regarding the second theme in this section, “challenges regarding working with different stakeholders”, the two key stakeholders mentioned were tourists and veterinarians. Challenges regarding the interface between tourists and working equines were described by several participants. One participant (P14) described the challenges a riding holiday tour operator had experienced associated with the views and “needs” of some of their tourist clients. For example, European tourists have expectations regarding the equipment that should be used (e.g., bits), and so, the tour operator adopted the use of equipment they would not usually use so that clients’ expectations were met. She described how, on one occasion, the operator changed the horse allocated to the rider to avoid conflict due to the rider’s method of handling a “slow” horse, an example of addressing cultural differences sensitively for the welfare of the horse. 

P5 explained that, in a riding stable for tourists, common practices have included tying horses up in the sun and hitting them as a means of control. Tourists had sometimes complained to the welfare organisation, who had passed on their concerns, but the owners did not always respond positively: *“some people think the pony is like a robot”* (P5). P10 described challenges due to differences in cultures between tourists and people providing horses to be used to climb a volcano (Guatemala) or be ridden on the beach (Panama). In both cases, tourists have regularly complained to the hotels about the conditions of the horses: *“You can really see the disconnect between how we feel about horses and how the owners feel about them”* (P10). 

P4 mentioned that this pressure from tourists has sometimes resulted in negative outcomes for the animals: *“With tourists, there’s a lot of righteousness and thoughts that are very judgmental. Some of them are justified and some of them are not. For example, the Thai pony is small, no more than 13 hands, but when fed well, in good body condition, and with good equipment can pull more than twice its weight. When tourists come and complain that the ponies are too small to be pulling the carts, it puts pressure on the owners to look for larger ponies, crossbreeds and breeds that are not suited to working in a hot climate, and they suffer as they cannot withstand the heat. So, tourists putting pressure on the operators without understanding the context, is a huge challenge.”*.

P1 described challenges that her organisation faces with the behaviour of expatriates who shout abuse at donkey cart drivers for beating the donkeys: *“If I was just riding my horse along the road and somebody started shouting and screaming at me, what you immediately do is either take it out on your donkey or get angry and be rude to the person shouting at you. It doesn’t help the situation.”*.

Several participants outlined challenges associated with veterinarians working cross-culturally. For example, P1 described *“What I say to all the vets that come out is that if all you can do is administer pain relief and give a kind word when that animal is dying, you’ve given more than it would’ve had if you hadn’t been there. A lot of them get very emotional and say “If I was in the UK I could do this, that, and the other”. And you can’t, you have to accept that, and you have to see what you can do in the situation with the resources you have.”* Similarly, P5 described that, sometimes, international veterinarans who have visited his project in Cambodia have contradicted each other regarding the way they do things. For example, one veterinarian said that there was no need to give antibiotics after castration, and the animal then developed an infection. P4 outlined challenges with Western professionals who have visited the projects she is involved with who have seemed to want to save the world and how such judgement is not conducive to working in a culturally sensitive way. *“I don’t want to kill the passion people feel that they can contribute to the world, but that’s the key word, contribute. It’s not saving the world. And we need to find a way to elevate a community without making them wrong all the time. We’re not always right.”* P4 also outlined the challenges this has posed for the in-country veterinary profession: *“People parachuting in creates a double standard for the veterinary profession that works within that country and that community, which makes matters worse because the profession needs to be able to sustain itself and grow and develop.”*.

### 3.4. Embracing Cultural Sensitivity

Two key themes emerged when the interviewees reflected on embracing cultural sensitivity: “the importance of developing relationships based in trust and respect between project staff and equine owners” and “the importance of working with local partners”. 

Regarding theme 1, P4 reflected that, *“Imposing western ideas on how things should be done and trying to bulldoze that concept or that idea to get to an action destroys the relationship or sometimes the work of an organization”.* Several participants outlined specific ways in which people show respect. For example, P6 explained *“In our culture […] you cannot say, “You have to do that”. We talk very gently, smoothly, and with respect. Many of our visitors keep smiling. This is very important, basic and fundamental. […] Regarding the intercultural differences and how we got accepted, the only way is that you need to be very, very respectful.”* P9 highlighted the importance of respect when working with horse owners who can sometimes be very shy and have self-confidence and self-esteem issues due to the low status of their work. A final example quote for this theme illustrates the importance of a respectful approach: *“I was talking to a group of elders about it and I said, how 10 years ago if you’d suggested euthanasia, everybody would say no. But now it’s, it’s happening. And they said, “One, you’ve always respected us in our beliefs and so on; and two, because you always try, we know that if we take our animal to horse and donkey, they will try everything, and if they can’t save it, no one can save it.” I’d never thought about it like that, it was amazing. So inadvertently that wasn’t the plan. It has kind of changed a culture, just by having respect for their beliefs.”* (P1).

Theme 2 (the importance of working with local partners and community representatives) was highlighted by all of the interviewees. P5 explained the approach used in the project he manages: *“When we work in a new community, we ensure we have a contact person or the leader of the pony owner group, to introduce us. We have found that where we have such contacts it is easy to build trust, but if we don’t get introduced to community, it is very difficult to step in.”* P2 also described working with carefully chosen partners: *“They speak the language, they understand the environment that they’re working in and are used to challenges of working there. They understand how traditional cultures work, how to negotiate, and how to work constructively in these areas.”.* P11 explained that it is vital to work with community leaders or with people who can “translate” your messages into not just the local language but ensure that it is framed in a culturally appropriate way as well: *“They listen to their peers more than they would listen to an instructor that’s come in, even from the next town”.*

P2 described the challenges in building genuine relationships with people: *“In Southern Africa, particularly in the rural projects where the mode of delivery is mostly primary healthcare clinics, people attend the clinics, and you don’t know if you will ever see them again or if you’ve seen them before.”* To address this, the projects she is involved with are working through intermediaries, representatives of communities that they have more contact with and support closely, recognising that they are on the ground 24/7, as opposed to the project staff who live 400 km away. 

P4 described how, in Thailand, her project facilitates different communities in interacting with each other and that by doing so she has observed camaraderie, exchanges of ideas, competition and positive outcomes: *“I think that in any culture, not just Thai culture, the power of community is very important because then you can drive action easier. When we had communities that were not doing so well, we invited representatives from communities that are doing well, to visit them and work with the ponies, do some assessment and talk about it. This worked—the first community got the message because they actually saw improvements could happen, it’s possible, as it has happened in a village not far from where they are.”*

P2 described how organisational culture is also relevant — the importance of recognising that organisations have specific cultures that, in partnership projects, need to be navigated for effective collaboration. She presented the example of a partner organisation with a very bureaucratic culture that stifled the activities on the ground at the community level. She also described how the teams in different countries of the international funding organisation she is working in all work in slightly different ways: *“I sometimes joke with the UK staff because sometimes British people will want to say something or assert themselves, but they want to be culturally sensitive—and what they are trying to say just goes straight over the heads of some of the project staff. I have to translate English into English and it’s nobody’s fault, it’s just getting used to each other.”*

## 4. Discussion

Each of the four key research objectives outlined in the introduction were met. The results of the thematic analysis, together with the summaries of the responses to each interview question, provide insight into the challenges and successful approaches regarding working cross-culturally to improve equine welfare. Throughout the interviews, responses from the different professionals were consistent, as were the case studies they described with shared key elements. 

The responses supported Saville-Troike’s findings regarding cross-cultural communication — that for successful communication to take place between people of different cultures, a person must have the appropriate linguistic knowledge, interaction skills and cultural knowledge [10]. 

Several participants expressed that the key to working cross-culturally is also the key to being socially literate in general. For example, P1 said *“It’s not really about the culture, it’s more about really common human principles that go across any situation, whether they’re people in your own culture or in other cultures. There’s so much that’s similar.”*. The prevalence of reflections on the importance of trust and respect also seem to be key to social interactions whether within or between cultures. 

Inherent in more general social literacy, is the need for perspective taking and an ability to view animal welfare from the position of the animal owners. Whether deliberately implementing perspective taking or doing so intuitively, all participants commented on the need to see situations from a local and cultural perspective, from recognising the interconnectedness of poverty and animal welfare to having an understanding of the religious barriers to a Western notion of welfare. This is in contrast with overt judgement by well-meaning, concerned tourists putting pressure on equid owners and inadvertently causing bad feelings that do not help the animals. Such concerns from Western tourists also suggests a “us against them” mentality regarding animal welfare in developing countries, focusing on a narrow range of welfare indicators (such as load size or condition). Although their concerns might be justified, the baseline for welfare should not be arbitrary factors but an established model such as the Five Domains [26]. In that case, Western standards of welfare can also be criticised for high numbers of horses experiencing pain (e.g. [27]), being unable to engage in their full ethogram of normal behaviours [28] and experiencing unresolved stress [29]. Thus, it is likely that cultural bias plays a key role in the perception of what constitutes “welfare”. 

P12 linked the benefits of working cross-culturally with the associated ethical responsibilities, which is certainly pertinent for anyone working on projects such as those described in this paper: *“I think working cross-culture is really enriching for us. You get to learn a lot, but I think we always have to take into account how it can be enriching for both sides and not just an extraction of information and then leaving the communities alone.”.* This was also reflected by P1: *“The international vets want to put everything to sleep, they don’t like the idea of waiting and seeing. One of the things we learned, is that a lot of our preconceptions are wrong. Many of the animals that I would’ve sworn could not get better have got better because we couldn’t put them to sleep—so we’ve learned a lot as well.”.*

As outlined in the introduction, Stanton’s work with regard to qualitative interview research described that, to avoid misunderstandings, the interviewer must try to walk in the other person’s shoes [11]. This is relevant to the present study not only in terms of communication between the professionals working cross-culturally with equid owners (arguably often in a similar context to that of an interviewer or researcher) but also regarding the cross-cultural communication between the researcher and interviewees. The sampling method, in retrospect, served to ensure that communication between the interviewer and interviewee was based on trust and respect due to either previous professional interactions or a personal introduction from shared contacts that was done in a very open and approachable way. On a related note, the cultural background of the interviewer in this study, in relation to the culture of the interviewees, should also be considered [30]. Reflexive practice [31] was used by the interviewer to self-consiously critique and evaluate how their subjectivity and own background might be influencing the research process.

A limitation of the study was the small number of interviewees. As explained in the methodology, the authors wanted to limit participation to enable a manageable dataset for a qualitative analysis. Further work could be done exploring professionals from a greater number of countries and, therefore, cultures. 

The study highlighted several areas for future research. It would be interesting to assess whether the perceptions of a greater number of interviewees across a greater range of countries follow the same themes and highlight the same topics as this study. It would also be interesting to gain a wider cultural representation, not just nationality, among the researchers themselves. In addition, different ways of defining culture considering the work by Weary and Robbins, which showed cultural variations in animal welfare conceptions even within communities, could be explored [32]. A deeper investigation into the details regarding how to work in a culturally sensitive way would generate more information that could inform projects. 

Recommendations for the practical application of this research in the field could include the development and testing of guidance to aid organisations and individuals who are working cross-culturally in projects such as those described in this work or in other animal welfare areas. This could be operationalised as, for example, a checklist of things to research before starting the work and ensuring the people are sufficiently trained in the skills needed for effective cross-cultural work (e.g., interpersonal skills, how to take an anthropological approach to asking questions by having an inquisitive mind and testing assumptions). Given the theme regarding the importance of working with local partners, a cocreation approach to project delivery, where partners in the focus communities are truly involved in project design and delivery, could be embedded in organisational guidelines or policies. Widespread adoption of these suggestions would enable the sector to truly evolve and avoid limitations and negative outcomes perhaps experienced in the past. 

This study is the first of its kind to examine the cross-cultural interpersonal aspects of equine welfare professionals. It enables an increased understanding of the experiences of others and provide valuable information for students and professionals.

## 5. Conclusions

The study provides an informed insight into the cross-cultural challenges and successful approaches used in international equine welfare projects. Qualitative research has resulted in the documentation of a range of perspectives and experiences that can be shared between professionals in this field to improve the impact of multi-stakeholder projects. The synthesis of experience and reflection, together with the information gained regarding lessons learnt about cross-cultural working in this field, can be operationalised to provide practical tools.

## Data Availability

Anonymised transcripts of the interviews are available from the authors.
